# Factors Associated With 10-Year Declines in Physical Health and Function Among Women During Midlife

**DOI:** 10.1001/jamanetworkopen.2021.42773

**Published:** 2022-01-10

**Authors:** Daniel H. Solomon, Alicia Colvin, Brittney S. Lange-Maia, Carol Derby, Sheila Dugan, Elizabeth A. Jackson, Kristine Ruppert, Carrie Karvonen-Gutierrez, Leah Santacroce, Elsa S. Strotmeyer, Nancy E. Avis

**Affiliations:** 1 Division of Rheumatology, Brigham and Women’s Hospital, Harvard Medical School, Boston, Massachusetts; 2 Department of Epidemiology, University of Pittsburgh, Pittsburgh, Pennsylvania; 3 Rush Alzheimer’s Disease Center and Department of Preventive Medicine, Rush University Medical Center, Chicago, Illinois; 4 Neurology, and Epidemiology and Population Health, Albert Einstein College of Medicine, Bronx, New York; 5 Department of Physical Medicine and Rehabilitation, Rush University Medical Center, Chicago, Illinois; 6 Department of Preventive Medicine, Rush University Medical Center, Chicago, Illinois; 7 Department of Medicine, University of Alabama at Birmingham, Birmingham; 8 School of Public Health, Department of Epidemiology, University of Michigan, Ann Arbor; 9 Department of Social Sciences & Health Policy, Wake Forest School of Medicine, Winston-Salem, North Carolina

## Abstract

**Importance:**

Women in midlife often develop chronic conditions and experience declines in physical health and function. Identifying factors associated with declines in physical health and function among these women may allow for targeted interventions.

**Objective:**

To examine the factors associated with clinically important 10-year declines in the physical component summary score (PCS) of the Short Form 36 (SF-36), a widely used patient-reported outcome measure, in women in midlife.

**Design, Setting, and Participants:**

This longitudinal cohort study collected data from geographically dispersed sites in the US. Participants were part of the Study of Women’s Health Across the Nation (SWAN), a racially and ethnically diverse cohort of women enrolled at or immediately before the menopause transition. Women have been followed for up to 21 years, between 1996 and 2016, with annual visits. Data were analyzed from October 2020 to March 2021.

**Exposures:**

Demographic indicators, health status measures, and laboratory and imaging assessments.

**Main Outcomes and Measures:**

The main outcome was a clinically important decline (≥8 points) on the PCS, based on the 10-year difference in scores between ages 55 and 65 years.

**Results:**

From the SWAN cohort of 3302 women, 1091 women (median [IQR] age, 54.8 [54.3-55.4] years; 264 [24.2%] Black women; 126 [11.6%] Chinese women; 135 [12.4%] Japanese women; 566 [51.9%] White women) were eligible for analyses based on duration of follow-up and availability of SF-36 data. At age 55, women had a median (IQR) body mass index (BMI; calculated as weight in kilograms divided by height in meters squared) of 27.0 (23.2-32.6), a median (IQR) baseline PCS of 53.1 (46.8-56.7), 108 women (9.9%) were current smokers, and 938 women (86.3%) had at least 1 comorbidity. Between ages 55 and 65 years, the median (IQR) change in PCS was −1.02 (−6.11 to 2.53) points with 206 women (18.9%) experiencing declines of 8 points or more. In multivariable models, factors associated with clinically important decline included higher baseline PCS (odds ratio [OR], 1.08; 95% CI, 1.06-1.11), greater BMI (OR, 1.06; 95% CI, 1.03-1.09), less educational attainment (OR, 1.87; 95% CI, 1.32-2.65), current smoking (OR, 1.93; 95% CI, 1.14-3.26), osteoarthritis (OR, 1.46; 95% CI, 1.01-2.09), clinically significant depressive symptoms (OR, 2.03; 95% CI, 1.34-3.09), and cardiovascular disease (OR, 2.06; 95% CI, 1.26-3.36).

**Conclusions and Relevance:**

In this cohort study, clinically important declines in women’s physical health and function were relatively common between ages 55 and 65 years. Several variables associated with these declines were identified as potentially useful components in a clinical score identifying women at increased risk of physical health and functional declines.

## Key Points

**Question:**

What patient-level factors in midlife women are associated with clinically important declines in physical health and function?

**Findings:**

In this cohort study among 1091 women, 206 women experienced clinically important declines in the physical component summary score of the Short Form 36. Several variables had significant associations, including low baseline health, high body mass index, less educational attainment, current smoking, and several comorbid conditions.

**Meaning:**

These findings suggest that patient variables could be useful for targeting interventions for women in midlife who are likely to experience clinically important declines in health and function in later life.

## Teaser

This cohort study examines factors associated with clinically important 10-year declines in the physical health of US women from age 55 to 65 years.

## Introduction

Identification of risk factors for declines in physical health and function could allow targeting prevention strategies to at-risk subgroups. Research suggests that declines in health and function that are common in later life can begin in midlife^1^ and that midlife factors may be associated with important health and functional declines in later life.^2^ Twenty-year follow-up data from the United Kingdom demonstrated that engaging in poor health behaviors (eg, tobacco use, reduced physical activity) was associated with higher mortality risk,^3^ suggesting that there are likely modifiable risk factors for declines in physical health and function.

Several studies, including our previous work within the Study of Women’s Health Across the Nation (SWAN),^4,5^ have identified other factors associated with declines in physical health and function. These include older age, higher body mass index (BMI; calculated as weight in kilograms divided by height in meters squared), reduced physical activity, tobacco use, and sleep problems.^4,6^ Prior studies of midlife risk factors in other cohorts have been longitudinal in nature,^7,8^ but none, except for a study by Avis et al^4^ from the same cohort, have included a racial and ethnically diverse study sample, to our knowledge. Most studies have focused on older adults^9^ rather than people during the midlife.^10^

Identifying factors associated with midlife declines in physical health and function has potential benefits. These factors could be used in combination to develop a risk score that would identify patients who are likely to experience clinically important declines, which could aid clinicians in targeting patients for interventions. In addition, the specific risk factors would help identify potential targets for interventions. With this background in mind, we examined the risk factors associated with of physical health and functional declines using the Short Form 36 (SF-36) physical component summary (PCS) score^11^ in a large, diverse, longitudinal cohort of women in midlife. Our analyses build on the prior work in SWAN by Avis et al^4^ by focusing on 10-year declines in PCS, using a cutoff to identify meaningful decline, and including additional variables (eg, C-reactive protein [CRP] level, blood pressure, lean body mass) that might be risk factors associated with declines.

## Methods

This cohort study used data from SWAN, which was reviewed and approved by local institutional review boards at each of 7 participating sites across the US. Participating sites screened and recruited women from their respective communities, starting in 1996.^12^ All women gave written informed consent. The Partners Healthcare Human Research Committee waived the need for further review for this study because we used previously collected data. This report follows the Strengthening the Reporting of Observational Studies in Epidemiology (STROBE) reporting guideline for cohort studies.

### Study Population and Design

All women considered for inclusion were participants in SWAN, a multicenter, multiethnic, and multiracial longitudinal study developed to assess psychosocial, lifestyle, clinical, and biological changes occurring through the menopausal transition. Entry criteria included age 42 to 52 years; intact uterus and at least 1 ovary, not using exogenous hormones or pregnant, breastfeeding, or lactating at enrollment; at least 1 menstrual period in the 3 months prior to screening; and self-identified race or ethnicity as either Black, Chinese, Hispanic, Japanese, or White. The 7 sites each recruited White women and women from 1 other racial or ethnic group, resulting in an inception cohort of 3302 women.^13,14^ The SWAN site enrolling Hispanic women was temporarily closed around the time of this study’s baseline, and these women had too few follow-up visits to be considered for this study. Race and ethnicity data were collected and analyzed because of concerns regarding differences in health outcomes across these groups. The SWAN cohort baseline visit occurred in 1996 and 1997, and the cohort continued to be seen approximately annually for up to 15 visits through 2016. The analytic baseline visit was the visit when women were closest to age 55 years. If values were missing at the baseline visit, we imputed using the most proximal nonmissing values from other visits.

We were interested in the longitudinal association between a baseline set of factors assessed at or near age 55 years and 10-year declines in physical health and function. Ten years was chosen for 2 reasons: first, 10 years provides a reasonable timeline for considering interventions and second, the 10-year proportion of women reaching a clinically important change in the PCS score was similar to the 5-year proportion, suggesting a stable outcome. Baseline variables were not updated in the analyses during the follow-up period. We required women to have had at least 2 assessments of physical health and function, measured as the PCS score of the SF-36, at least 10 years apart. The measurements were required to be within 3 years of the woman’s 55th and 65th birthdays.

### Outcome Definition

The outcome of interest was clinically important change in the SF-36 PCS. The SF-36 is a generic health-related quality of life measure yielding 8 subscales and 2 summary scores; the PCS is 1 of the summary scores.^15^ Twenty items from the SF-36 comprise the PCS, which is calculated by standardizing each of the 8 SF-36 scales and weighting them using all 8 domains of the SF-36 scales (eTable 1 in the Supplement). The SF-36 was administered at SWAN visits 6, 8, 10, 12, 13, and 15. We chose the SF-36 assessments closest to a woman’s 55th birthday as her baseline assessment and the visit closest to a woman’s 65th birthday as her follow-up. The primary outcome was the 10-year change in PCS between ages 55 and 65 years.

The 10-year change in PCS was further categorized into clinically important decline, no clinically important change, or improvement. The minimally clinically important difference for the PCS varies by condition, but it is generally between 5 and 10 points.^16,17^ We chose an 8-point difference as the primary definition of a clinically important difference.^18^ We also predefined 2 secondary outcomes: the 5-year change in PCS between ages 60 and 65 years and a 6-point difference in 10-year PCS.

### Potential Associations

We defined factors potentially associated with outcomes using the information reported during the SWAN annual visits. Age in years was calculated at each SWAN visit and updated in the analyses. Race and ethnicity were self-identified at a screening interview prior to baseline. Participants who identified as Mexican or Mexican American, mixed race or ethnicity, or other race or ethnicity were not included in SWAN.^19^ Educational attainment categories were collected only at the SWAN baseline and included less than a high school degree, high school degree, college degree, and postcollege degree. Variables were collected throughout SWAN visits and the measure closest to age 55 years (or 60 years in secondary analyses) was used. Menopausal status was based on self-reported bleeding patterns and categorized premenopausal, early perimenopausal, late perimenopausal, postmenopausal (natural), postmenopausal (bilateral oophorectomy), unknown using hormone treatment, and unknown after hysterectomy. Marital status was categorized as never married, married or living as married, separated, divorced, or widowed. Alcohol use was determined via questionnaire and was categorized as less than 1 drink per week, 1 to 7 drinks per week, greater than 7 drinks per week, or missing. Smoking status included never, past, or current. Medical insurance at SWAN baseline included only 2 categories: some medical insurance (public or private) or no medical insurance. Relevant major medical conditions were defined using self-reported physician-diagnosed information from participants. Chronic conditions were considered present from the time they were self-reported through the end of follow-up, including thyroid disease, osteoarthritis, osteoporosis, diabetes, cardiovascular disease (myocardial infarction, stroke, or angina), hypertension, hyperlipidemia, cancer, and venous thromboembolic disease. Presence of depressive symptoms was defined using a score of 16 or greater on the Center for Epidemiologic Study–Depression (CES-D) scale. BMI was calculated from objectively measured height and weight. Sleep disturbance was defined as at least 3 or more nights per week of difficulty initiating sleep, difficulty remaining asleep, or early morning awakenings.^20^ Prescription medications reported to be used regularly by women were included as a count variable. Finally, several aspects of the physical examination, physical function, or laboratory measures were examined. These include the total score on the Kaiser Physical Activity Survey,^21^ the PCS at age 55 years, systolic and diastolic blood pressure, high-sensitivity C-reactive protein (hsCRP; Dade-Behring) level, and skeletal muscle mass. Skeletal muscle mass was estimated using bioelectrical impedance based on whole body muscle mass using previously published equations, including height, conductance, sex, and age^22^; this unitless measure is then standardized by dividing it by the square root of height.

### Statistical Analyses

The first step was to categorize women according to their PCS change score: improved, declined, and no change. Because we were most interested in identifying risk of decline, we combined the no change and improved groups. We used χ^2^ tests or *t* tests to compare variables across these groups. The median starting values at age 55 years and final values at age 65 years were examined for the PCS categories.

The primary analyses examined the factors associated with 10-year clinically important declines in the PCS. Thus, logistic regression models were examined to evaluate the associations between participant characteristics measured at age 55 years or before (independent variables) and 10-year clinically important decline in PCS (dependent variable); the reference group for PCS included women who had a stable or improving PCS. We first assessed the univariate associations and then constructed multivariable logistic regression models. All variables with *P* < .10 were included in the multivariable models. Race and ethnicity were forced into the final multivariable models since a main thrust of SWAN is the examination of racial and ethnic differences in the health of women in the midlife.

Model fit characteristics were evaluated for all multivariable models. These included the area under the receiver operating characteristic curve (AUROC, derived from the *C* statistic, a measure of model discrimination), Akaike information criterion (in which lower is better), and bayesian information criterion (in which lower is better). A sensitivity survival analysis was conducted to assess variables associated with the time until reaching the primary outcome; a Cox proportional hazards regression was estimated for the survival analysis.

Analyses were carried out using SAS statistical software version 9.4 (SAS Institute), and graphs were created in R version 4.0.3 (R Project for Statistical Computing). *P* values were 2-sided, and statistical significance was set at *P* < .05. Data were analyzed from October 2020 to March 2021.

## Results

**Figure 1.**
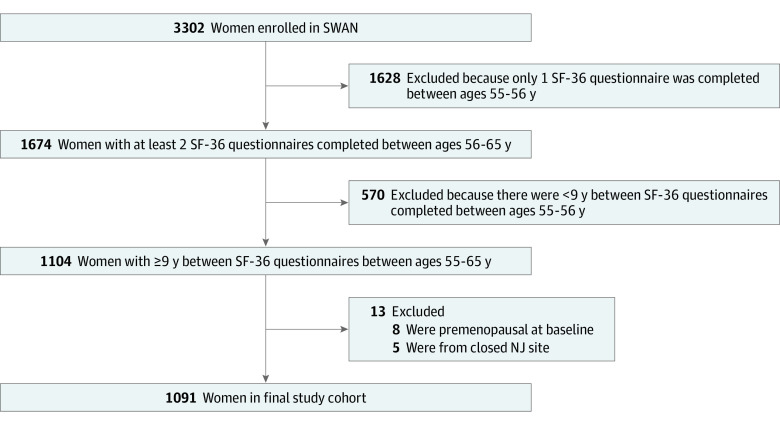
Cohort Allocation Flowchart NJ indicates New Jersey; SF-36, Short Form 36; and SWAN, Study of Women’s Health Across the Nation.

Of 3302 women enrolled in SWAN, 1091 were included in these analyses (Figure 1). Characteristics of women included in the analyses are shown in Table 1. At the visit closest to age 55, the sample had a median (IQR) age of 54.8 (54.3-55.4) years and a median BMI of 27.0 (23.2-32.6). Approximately one-quarter of women identified as Black (264 women [24.2%]), 126 (11.6%) identified as Chinese, 135 (12.4%) identified as Japanese, and half of the women identified as White (566 women [51.9%]). Two-thirds of women had experienced natural menopause by age 55 years. Two-thirds of women used no alcohol or consumed less than 1 drink per week. Only 10% of women were current smokers (108 women [9.9%]) and 938 women (86.3%) reported at least 1 major comorbidity, with hyperlipidemia (579 women [53.1%]), osteoarthritis (536 women [49.1%]), and hypertension (472 women [43.4%]) being most common. Women had relatively good physical activity at baseline, with a median (IQR) Kaiser Physical Activity Score of 7.6 (6.2-8.8) at baseline. Baseline median (IQR) PCS score was 53.1 (46.8-56.7).

**Table 1.** Characteristics of Participants at Age 55 Years

Characteristic	No. (%)			*P* value
	Total cohort (N = 1091)^a^	10-Year change in physical component score		
		≥8-point decline (n = 206)	Increase or no significant change (n = 885)	
Age, median (IQR), y	54.8 (54.3-55.4)	54.7 (54.3-55.4)	54.8 (54.3-55.4)	.94
BMI, median (IQR)	27.0 (23.2-32.6)	29.6 (24.7-35.6)	26.3 (22.9-31.6)	<.001
No health insurance	40 (3.7)	12 (5.8)	26 (3.3)	.06
Race or ethnicity^b^				
Black	264 (24.2)	65 (31.6)	199 (22.5)	.02
Chinese	126 (11.6)	22 (10.7)	104 (11.8)	
Japanese	135 (12.4)	16 (7.8)	119 (13.5)	
White	566 (51.9)	103 (50.0)	463 (52.3)	
Menopausal status				
Postmenopausal				.75
Surgical	56 (5.0)	10 (4.9)	44 (5.0)	
Natural	733 (67.3)	141 (68.5)	592 (67.0)	
Perimenopausal				
Late	111 (10.2)	18 (8.7)	93 (10.5)	
Early	119 (10.9)	26 (12.6)	93 (10.5)	
Unknown				
Hormone therapy	50 (4.6)	9 (4.4)	41 (4.6)	
Posthysterectomy	22 (2.2)	2 (1.0)	20 (2.3)	
Smoking status				
Never	669 (61.4)	109 (52.9)	560 (63.4)	.003
Current	108 (9.9)	32 (15.5)	76 (8.6)	
Past	312 (28.7)	65 (31.6)	247 (28.0)	
Alcohol use, drinks/wk				
None	460 (42.2)	99 (48.1)	361 (40.8)	.12
<1	269 (24.7)	39 (18.9)	230 (26.0)	
1-7	187 (17.1)	31 (15.1)	156 (17.6)	
>7	75 (6.9)	14 (6.8)	61 (6.9)	
No answer given	100 (9.2)	23 (11.2)	77 (8.7)	
Education				
≥College	550 (50.6)	76 (36.9)	474 (53.8)	<.001
≤High school	537 (49.4)	130 (63.1)	407 (46.2)	
Difficulty paying for basics	245 (22.5)	64 (31.1)	181 (20.5)	.001
Sleep disturbance^c^	500 (45.8)	105 (51.0)	395 (44.6)	.10
Comorbid conditions present				
Diabetes	163 (14.9)	40 (19.4)	123 (13.9)	.045
Hypertension	473 (43.4)	108 (52.4)	365 (41.2)	.006
Hyperlipidemia	579 (53.1)	118 (57.3)	461 (52.1)	.18
Cardiovascular disease	142 (13.0)	40 (19.42)	102 (11.53)	.002
Osteoarthritis	536 (49.1)	116 (56.3)	420 (47.5)	.02
Osteoporosis	181 (16.6)	23 (11.2)	158 (17.6)	.02
Thyroid disease	272 (24.9)	55 (26.7)	217 (24.5)	.51
Cancer	68 (6.2)	13 (6.3)	55 (6.2)	.96
Depressive symptoms^d^	165 (15.1)	47 (22.8)	118 (13.3)	<.001
Physical component score, median (IQR)	53.1 (46.8-56.7)	53.3 (48.2-57.2)	53.1 (46.6-56.6)	.02
Kaiser Physical Activity Score, median (IQR)	7.6 (6.2-8.8)	7.3 (5.9-8.6)	7.6 (6.3-8.9)	.006
Blood pressure, median (IQR), mm Hg				
Systolic	115 (105-126)	119 (107-129)	114 (105-125)	.008
Diastolic	73 (67-80)	75 (68-80)	73 (67-80)	.19
hsCRP, median (IQR), mg/L	1.7 (0.6-5.3)	2.4 (0.9-6.8)	1.6 (0.6-4.8)	.04
Skeletal muscle mass, median (IQR)^e^	1.6 (1.4-1.7)	1.6 (1.5-1.8)	1.6 (1.4-1.7)	.001
Prescription medications used, median (IQR), No.	2.0 (0.0-3.0)	2.0 (1.0-4.0)	2.0 (0.0-3.0)	.08

Abbreviations: BMI, body mass index (calculated as weight in kilograms divided by height in meters squared); hsCRP, high-sensitivity C-reactive protein.

^a^ Missing data included 2 participants for smoking status, 100 participants for alcohol use, 2 participants for insurance status, 4 participants for education, 2 participants for Kaiser Physical Activity Score, 1 participant for hsCRP, and 2 participants for BMI.

^b^ The race and ethnicity categories were developed in the 1990s and do not adequately characterize all Chinese and Japanese ethnicities.

^c^ Sleep disturbance was defined as at least 3 nights per week of difficulty initiating sleep, difficulty remaining asleep, or early morning awakenings.

^d^ Depressive symptoms were defined as a score of 16 or greater on the Center for Epidemiology Study–Depression scale.

^e^ Skeletal muscle mass is a unitless quotient estimated in SWAN using bioelectrical impedance based on whole body muscle mass using previously published equations including height, conductance, sex, and age; this unitless measure is then standardized by dividing it by the square root of height.

SWAN cohort women excluded from this study were compared with the study population (eTable 2 in the Supplement). While there were many statistically significant differences, the important differences between excluded and included women included BMI, insurance coverage, tobacco use, comorbidities, and race and ethnicity.

**Figure 2.**
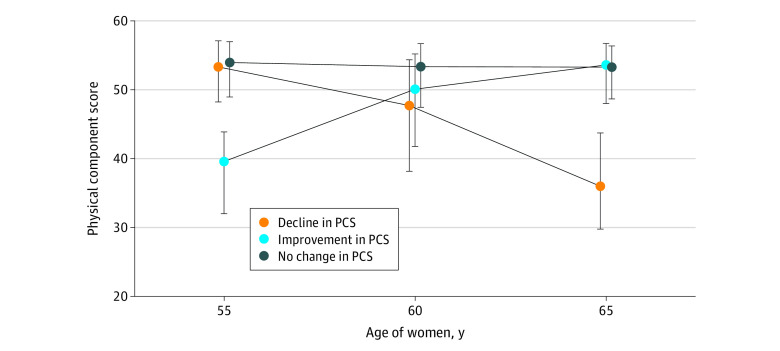
Baseline and Follow-up Physical Component Scores (PCS) for Women Who Experienced Significant Declines, No Change, or Improvements Dots indicate medians; whiskers, IQRs.

Based on their 10-year change in PCS, 206 women (18.9%) experienced a clinically important decline of at least 8 points, 791 women (72.5%) experienced no clinically important change, and 94 women (8.6%) experienced a clinically important improvement. The median PCS changes for the 3 groups of women are shown in Figure 2.

Women who experienced a clinically important decline differed on many characteristics at age 55 years compared with women who improved or showed no change. Women who experienced a decline were more likely to be Black and less likely to be Japanese (Table 1). They had higher BMI, were more likely to smoke, were less likely to have attended college, and had more trouble paying for basics. They also had more comorbidities (namely, diabetes, hypertension, cardiovascular disease, osteoarthritis, and osteoporosis), were more likely to have clinically significant depressive symptoms, and reported less physical activity. They also differed on physical measurements, with higher systolic blood pressure and higher hsCRP levels (Table 1).

Each of the potentially associated factors was examined in bivariate logistic regression, and variables with *P* < .10 were advanced to multivariable models. Race and ethnicity was added into the model (Table 2). In multivariable models, variables associated with clinically important declines in PCS included higher baseline PCS (odds ratio [OR], 1.08; 95% CI, 1.06-1.11), greater BMI (OR, 1.07; 95% CI, 1.03-1.12), less educational attainment (OR, 1.87; 95% CI, 1.32-2.65), current smoking (OR, 1.93; 95% CI, 1.14-3.26), osteoarthritis (OR, 1.46; 95% CI, 1.01-2.09), having clinically significant depressive symptoms (OR, 2.03; 95% CI, 1.34-3.09), and cardiovascular disease (OR, 2.06; 95% CI, 1.26-3.36). Although Black women were more likely to be in the declining group than White women in the bivariate analyses, Black race was no longer significant in the multivariable model. The AUROC was 0.73, suggesting moderate model discrimination. An additional model retained only the variables with ORs and CIs that excluded 1.00 (Table 2).

**Table 2.** Bivariate and Multivariable Associations Between Characteristics at Age 55 Years and 10-Year Declines in Physical Component Score

Characteristic at age 55 y	Odds ratio (95% CI)^a^		
	Bivariate	Multivariable (fully adjusted)	Multivariable (only significant)
BMI, per 1-unit increase	1.05 (1.03-1.07)	1.06 (1.03-1.09)	1.06 (1.04-1.09)
Health insurance status			
Insured	1 [Reference]	1 [Reference]	NA^b^
Uninsured	1.89 (0.94-3.78)	1.23 (0.55-2.74)	NA^b^
Race or ethnicity^c^			
White	1 [Reference]	1 [Reference]	NA^b^
Black	1.47 (1.03-2.09)	1.02 (0.68-1.54)	NA^b^
Chinese	0.95 (0.57-1.58)	1.68 (0.92-3.07)	NA^b^
Japanese	0.60 (0.34-1.06)	0.86 (0.45-1.61)	NA^b^
Menopausal status			
Post-menopausal			
Natural	1 [Reference]	NA^b^	NA^b^
Surgical	0.95 (0.47-1.94)	NA^b^	NA^b^
Perimenopausal			
Late	0.81 (0.48-1.39)	NA^b^	NA^b^
Early	1.17 (0.73-1.88)	NA^b^	NA^b^
Unknown			
Menopause, hormone therapy	0.92 (0.44-1.94)	NA^b^	NA^b^
Posthysterectomy	0.42 (0.10-1.82)	NA^b^	NA^b^
Smoking status			
Never	1 [Reference]	1 [Reference]	1 [Reference]
Past	1.35 (0.96-1.90)	1.48 (1.00-2.17)	1.29 (0.90-1.84)
Current	2.16 (1.36-3.43)	1.93 (1.14-3.26)	1.92 (1.17-3.15)
Alcohol use, drinks/wk			
None	1.62 (1.08-2.43)	1.37 (0.88-2.16)	NA^b^
<1	1 [Reference]	1 [Reference]	NA^b^
1-7	1.17 (0.70-1.96)	1.25 (0.72-2.16)	NA^b^
>7	1.35 (0.69-2.65)	1.29 (0.62-2.66)	NA^b^
No answer given	1.76 (0.99-3.13)	1.78 (0.95-3.32)	NA^b^
Education			
≥College	1 [Reference]	1 [Reference]	1 [Reference]
≤High school	1.99 (1.46-2.72)	1.87 (1.32-2.65)	2.00 (1.43-2.79)
Difficulty paying for basics	1.75 (1.25-2.46)	1.42 (0.95-2.10)	NA^b^
Sleep disturbance	1.29 (0.95-1.75)	NA^b^	NA^b^
Comorbid conditions present^d^			
Diabetes	1.49 (1.01-2.22)	0.79 (0.45-1.30)	NA^b^
Hypertension	1.57 (1.16-2.13)	1.10 (0.76-1.60)	NA^b^
Hyperlipidemia	1.23 (0.91-1.68)	NA^b^	NA^b^
Cardiovascular disease	1.85 (1.24-2.77)	2.06 (1.26-3.36)	2.01 (1.27-3.18)
Osteoarthritis	1.43 (1.05-1.94)	1.46 (1.01-2.09)	1.46 (1.03-2.08)
Osteoporosis	0.58 (0.36-0.92)	0.49 (0.29-0.83)	NA^b^
Thyroid disease	1.12 (0.79-1.58)	NA^b^	NA^b^
Cancer	1.02 (0.54-1.90)	NA^b^	NA^b^
Depressive symptoms^e^	1.92 (1.32-2.81)	2.03 (1.34-3.09)	2.06 (1.37-3.10)
Physical component score, per 1-unit increase	1.02 (1.00-1.04)	1.08 (1.06-1.11)	1.07 (1.04-1.09)
Kaiser Physical Activity Score, per 1-unit increase	0.89 (0.81-0.97)	0.92 (0.83-1.02)	NA^b^
hsCRP, per 1 mg/L increase	1.02 (1.00-1.03)	0.99 (0.97-1.01)	NA^b^
Skeletal muscle mass, per 1-unit increase^f^	2.79 (1.48-5.26)	NA	NA^b^
No. of prescription medications, per 1-unit increase	1.05 (0.99-1.10)	1.07 (0.99-1.14)	NA^b^
Model fit statistics			
AUROC	NA	0.7314	0.711
AIC	NA	1054.134	1056.247
BIC	NA	1059.118	1061.236

Abbreviations: AIC, Akaike information criterion; AUROC, area under the receiver operating characteristic curve from the *C* statistic; BIC, bayesian information criterion; BMI, body mass index (calculated as weight in kilograms divided by height in meters squared); hsCRP, high-sensitivity C-reactive protein; NA, not applicable.

^a^ Reference group is women without PCS decline, unless otherwise noted. Variables were selected for the multivariable models based on *P* < .10 in bivariate logistic regression. The multivariable (significant only) model includes only the variables in the fully adjusted model with 95% CIs that excluded 1. Additionally, race/ethnicity were forced into all multivariable models.

^b^ Variable not advanced to the multivariable models.

^c^ The race and ethnicity categories were developed in the 1990s and do not adequately characterize all Chinese and Japanese ethnicities.

^d^ For each comorbidity, the reference category was the comorbidity not present.

^e^ Depressive symptoms defined as a score of 16 or greater on the Center for Epidemiologic Studies–Depression scale.

^f^ Skeletal muscle mass was significant in bivariate analyses but had 4.8% missing values and was not advanced to multivariable analyses.

Variables from the multivariable models were also tested in secondary analyses (Table 3; eTable 3 in the Supplement). The sensitivity analyses assessing 5-year decline in PCS and 10-year decline of at least 6 points as dependent variables and a survival analysis found similar significant associations. However, both logistic models had worse model fit statistics compared with the primary 10-year analyses.

**Table 3.** Sensitivity Analyses for Multivariable Associations Between Women’s Characteristics and Declines in Physical Component Score

Characteristic at age 55 y	Odds ratio (95% CI)	
	Association with 5-y change in PCS (n = 945)^a^	Association with 10-y 6-point decline in PCS (n = 1091)
BMI, per 1-unit increase	1.03 (0.99-1.06)	1.05 (1.02-1.08)
Health insurance status		
Insured	1 [Reference]	1 [Reference]
Uninsured	1.85 (0.80-4.29)	1.58 (0.75-3.30)
Race/ethnicity^b^		
White	1 [Reference]	1 [Reference]
Black	1.08 (0.65-1.79)	1.05 (0.72-1.53)
Chinese	2.29 (1.14-4.61)	1.49 (0.87-2.55)
Japanese	1.58 (0.79-3.16)	0.88 (0.51-1.50)
Smoking status		
Never	1 [Reference]	1 [Reference]
Past	1.00 (0.64-1.57)	1.17 (0.83-1.65)
Current	0.46 (0.19-1.10)	1.53 (0.94-2.49)
Alcohol use, drinks/wk		
None	0.67 (0.41-1.08)	0.89 (0.60-1.30)
<1	1 [Reference]	1 [Ref]
1-7	1.25 (0.70-2.23)	0.95 (0.60-1.52)
>7	1.10 (0.51-2.37)	1.12 (0.60-2.07)
No answer given	NA	0.89 (0.50-1.58)
Education		
≥College	1 [Ref]	1 [Reference]
≤High school	0.98 (0.65-1.48)	1.57 (1.16-2.14)
Difficulty paying for basics	1.41 (0.88-2.25)	1.42 (0.95-2.10)
Comorbid conditions present		
Diabetes	1.40 (0.82-2.41)	0.56 (0.35-0.89)
Hypertension	1.16 (0.74-1.82)	1.04 (0.75-1.46)
Cardiovascular disease	1.75 (1.03-2.97)	2.11 (1.34-3.33)
Osteoarthritis	1.63 (1.06-2.50)	1.48 (1.07-2.04)
Osteoporosis	0.60 (0.34-1.07)	0.66 (0.42-1.02)
Depressive symptoms^c^	2.08 (1.27-3.41)	1.63 (1.10-2.41)
Physical component score, per 1-unit increase	1.07 (1.04-1.10)	1.08 (1.06-1.10)
Kaiser Physical Activity Score, per 1-unit increase	0.87 (0.78-0.98)	0.90 (0.82-0.99)
hsCRP, per 1-mg/L increase	0.98 (0.94-1.02)	1.01 (0.99-1.03)
No. of prescription medications, per 1-unit increase	1.09 (1.00-1.19)	1.04 (0.98-1.11)
Model fit statistics		
AUROC	0.7069	0.7000
AIC	759.367	1233.312
BIC	764.146	1238.296

Abbreviations: AIC, Akaike information criterion; AUROC, area under the receiver operating characteristic curve from the *C* statistic; BIC, bayesian information criterion; BMI, body mass index (calculated as weight in kilograms divided by height in meters squared); hsCRP, high-sensitivity C-reactive protein; PCS, physical component score.

^a^ Baseline variables measured at age 60 years. This model used a smaller sample size of 945 women, with 151 women meeting the 8-point decline outcome.

^b^ The race and ethnicity categories were developed in the 1990s and do not adequately characterize all Chinese and Japanese ethnicities.

^c^ Depressive symptoms defined as a score of 16 or greater on the Center for Epidemiologic Studies–Depression scale.

## Discussion

This cohort study examined 10-year declines in physical health and function among women in midlife. Among a longitudinal cohort of women followed for 10 years between ages 55 and 65 years, we found approximately 19% experienced clinically important declines in PCS. Characteristics at age 55 years that were significantly associated with these declines included higher baseline physical health and function, higher BMI, lower educational attainment, current smoking, osteoarthritis, cardiovascular disease, and clinically significant depressive symptoms. The multivariable model fit was good and consistent in sensitivity analyses.

Several studies have focused on analyzing midlife factors associated with improvements in quality of life.^4,8^ These studies found that better urinary function, a lack of sleep problems, fewer chronic health conditions, and improved social and psychological status were associated with better physical health and function. While some investigators have examined health-promoting behaviors, others have examined risky behaviors, such as smoking, poor diet, decreased physical activity, and high alcohol consumption.^3^ In an older population, the Health and Retirement Study^23^ also found that an increasing number of comorbid conditions was associated with a worse trajectory of physical function. Similar to this study, arthritis, cardiovascular disease, and depressive symptoms were among the comorbid conditions that were associated with risk of physical function decline. Our approach differed in that we examined whether midlife factors were associated with physical health and functional declines by age 65 years.

The implications for these findings are several. First, identification of risk factors associated with 10-year declines could be used in a clinical risk score. Risk scores have been used widely in clinical medicine to personalize management strategies and stratify risk. The most well known of these risk scores, the Framingham coronary heart disease prediction scores,^24^ are used in cardiovascular medicine and help target interventions. Our model fit statistics suggest that the variables we selected accurately identified women likely to have clinically important declines. If these variables are replicated in an external cohort, then a risk score for clinically important declines in midlife women could be pursued.

Second, characterizing the population of women who are likely to have clinically important declines in physical health and function should help early identification of modifiable factors. While some variables identified in the current analyses may not be easily modified (eg, educational attainment), others may be targeted for preventative or therapeutic interventions, including BMI, current smoking, and clinically significant depressive symptoms. More detailed analyses of populations at high risk of decline might identify other modifiable targets. Mediators of declines in physical health and function may differ from markers of women likely to experience such declines. Future analyses could examine differences in those who experience early vs late PCS decline and whether certain covariates may mediate changes in PCS.^25^

### Strengths and Limitations

This study has strengths and limitations. We examined a diverse, multiethnic group of women, but they may not be representative of all women.^25^ They were all from the US but only from 6 sites. There were differences noted between women who were included and those excluded, with the excluded participants having more comorbidities. The main reason for exclusion was the lack of evaluable SF-36 data in the correct time periods. This occurred mainly because women missed a study visit, declined completion of the SF-36, or were lost to follow-up. Subanalysis found that the included cohort was very similar to the total cohort in SWAN. In addition, there were some missing values at the baseline visit, but very few after imputing using the most proximal nonmissing values from other visits. Only 5 Hispanic women from 1 site had the required follow-up; they were excluded because of the very small sample of women of this ethnicity, limiting generalizability. Data across a relatively long follow-up were included, and a wide range of variables were considered. However, we did not have information on earlier life factors (eg, BMI), as well as many laboratory values (eg, hemoglobin, estimated glomerular filtration rate) that might also be associated with long-term health outcomes. We also did not have data on many psychological and social factors that have been associated with more healthful aging in prior work, including the Midlife in the United States study.^8^ While the SF-36 is a widely used patient-reported outcome measure, there are other health-related qualify of life measures.^26^

## Conclusions

This cohort study assessed the risk factors associated with clinically important declines in physical health and function in midlife women in the US. These declines are important among people in midlife and are associated with long-term health status concerns in older adults. While many health and social factors can lead to declines in physical health and function, there may be factors that can be modified in midlife women to prevent declines. Some have speculated that midlife could be a window of opportunity for long-term qualify of life improvement.^27^ Our data are observational, thus not permitting strong inferences about targets for interventions. If the variables we observed are found to be associated with physical health and functional declines in an external cohort, it may be worth constructing a risk score to identify women at high risk of clinically important decline, with the hope of identifying variables that could be mediated with interventions.

## Notes

Supplement. **eTable 1.** Scoring of the Physical Component Score of the SF-36 **eTable 2.** Baseline Characteristics at Entry Into SWAN of Included Compared With Excluded SWAN Participants **eTable 3.** Cox Proportional Hazard Regression Model Results **eAppendix.** The Study of Women's Health Across the Nation (SWAN) Contributors
