# Supplementary material for: Factors Associated With 10-Year Declines in Physical Health and Function Among Women During Midlife
Source: JAMA Netw Open. 2022 Jan 10;5(1):e2142773. doi: 10.1001/jamanetworkopen.2021.42773 (PMC8749479; doi:10.1001/jamanetworkopen.2021.42773)
Supplement: Supplement. — eTable 1. Scoring of the Physical Component Score of the SF-36 eTable 2. Baseline Characteristics at Entry Into SWAN of Included Compared With Excluded SWAN Participants eTable 3. Cox Proportional Hazard Regression Model Results eAppendix. The Study of Women's Health Across the Nation (SWAN) Contributors [file jamanetwopen-e2142773-s001.pdf]

Supplemental Online Content

Solomon DH, Colvin A, Lange-Maia BS, et al. Factors associated with 10-year declines in physical health and function among women during midlife. *JAMA Netw Open*. 2022;5(1):e2142773. doi:10.1001/jamanetworkopen.2021.42773

eTable 1. Scoring of the Physical Component Score of the SF-36
eTable 2. Baseline Characteristics at Entry Into SWAN of Included Compared With Excluded SWAN Participants
eTable 3. Cox Proportional Hazard Regression Model Results
eAppendix. The Study of Women's Health Across the Nation (SWAN) Contributors

This supplemental material has been provided by the authors to give readers additional information about their work.

© 2022 Solomon DH et al. *JAMA Network Open.*

eTable 1. Scoring of the Physical Component Score of the SF-36

SF-36 Domains	Orthogonal PCS
Physical Function	0.42
Role-Physical	0.35
Bodily Pain	0.32
General Health	0.25
Vitality	0.03
Social Functioning	-0.01
Role-Emotional	-0.19
Emotional Well-Being	-0.22

Adapted from Farivar SS, Cunningham WE, Hays, RD. Correlated physical and mental health summary scores for the SF-36 and SF-12 Health Survey. Health and Quality of Life Outcomes 2007;5:54.The PCS is computed using an aggregate physical component score from weighted z-scores, and then transforming the aggregate score to a norm-base score with a mean of 50 and standard deviation of 10, using the 1998 general U.S. population.

© 2022 Solomon DH et al. *JAMA Network Open.*

eTable 2. Baseline Characteristics at Entry Into SWAN of Included Compared With Excluded SWAN Participants

Characteristics at SWAN baseline	Included Women (n = 1091)	Excluded Women (n = 2211)	P value	Total SWAN Cohort
	N (%) or median (interquartile range)			
Age, years	46.6 (45.0, 48.3)	45.7 (43.6, 48.2)	<0.0001	46.2 (44.1, 48.3)
Body mass index, kg/m2	25.4 (22.2, 30.8)	27.3 (23.3, 32.6)	<0.0001	26.7 (22.9, 32.1)
Insurance status, none	40 (3.7)	233 (10.6)	<0.0001	273 (8.3)
Race or ethnicity			<0.0001	
Black	264 (24.2)	670 (30.3)		934 (28.3)
Chinese	126 (11.6)	124 (5.6)		250 (7.6)
Hispanic	0 (0.0)	286 (12.9)		286 (8.7)
Japanese	135 (12.4)	146 (6.6)		281 (8.5)
White	566 (51.9)	985 (44.6)		1551 (47.0)
Menopausal status			0.1555	
Early Peri	472 (43.4)	1029 (47.0)		1501 (45.8)
Pre	613 (56.4)	1157 (52.8)		1770 (54.0)
Unknown/HT	2 (0.2)	4 (0.2)		6 (0.2)
Smoking status, never	678 (62.1)	1212 (54.9)	<0.0001	1890 (57.3)
Past	280 (25.7)	550 (24.9)		830 (25.3)
Current	133 (12.2)	447 (20.2)		580 (17.6)
Alcohol use, none	506 (46.4)	1047 (47.4)	0.0021	1553 (47.0)
<1/week	90 (8.3)	236 (10.7)		326 (9.9)
1-7/week	290 (26.6)	523 (23.7)		813 (24.6)
>7/week	160 (14.7)	281 (12.7)		441 (13.4)
Missing	45 (4.1)	124 (5.6)		169 (5.1)
Education – College or graduate degree	500 (50.6)	851 (39.0)	<0.0001	1401 (42.8)
High school or less	537 (49.4)	1333 (61.0)		1870 (57.2)
Sleep Disturbance	277 (25.4)	665 (30.1)	0.0050	942 (28.5)
Diabetes	50 (4.6)	163 (7.4)	0.0021	213 (6.5)
Hypertension	198 (18.2)	476 (21.5)	0.0234	674 (20.4)
Hyperlipidemia	185 (16.9)	435 (19.7)	0.0600	620 (18.8)
CVD	25 (2.3)	124 (5.6)	<0.0001	149 (4.5)
Osteoarthritis	167 (15.3)	418 (18.9)	0.0109	585 (17.7)
Osteoporosis	18 (1.7)	65 (2.9)	0.0259	83 (2.5)
Thyroid disease	122 (11.2)	247 (11.2)	0.9925	369 (11.2)
Cancer	25 (2.3)	40 (1.8)	0.3687	65 (2.0)
Depression as defined by CES-D ≥ 16	228 (20.9)	576 (26.1)	0.0010	804 (24.4)
Kaiser Physical Activity Score	7.7 (6.6, 9.0)	7.5 (6.3, 8.8)	0.0015	7.6 (6.4, 8.9)
C-reactive protein	1.4 (0.5, 4.6)	2.2 (0.8, 6.6)	0.0004	1.8 (0.6, 5.9)

CES-D, Center for Epidemiologic Studies-Depression. NA, not available.

© 2022 Solomon DH et al. *JAMA Network Open.*

eTable 3. Cox Proportional Hazard Regression Model Results

**Characteristic at Age 55**	**Hazard Ratio (95% Confidence Interval)**
N with 8-point decline at any timepoint	681
BMI	1.02 (1.01, 1.03)
Education (Some college or more)	Reference
High School or less	1.13 (0.97, 1.32)
Osteoarthritis	1.30 (1.11, 1.53)
Cancer	1.24 (0.97, 1.58)
CES-D	1.56 (1.30, 1.88)
Kaiser Physical Activity Score	0.97 (0.93, 1.01)
CES-D, Center for Epidemiologic Studies-Depression	

© 2022 Solomon DH et al. *JAMA Network Open.*

eAppendix. The Study of Women's Health Across the Nation (SWAN) Contributors

Clinical Centers: University of Michigan, Ann Arbor – Siobán Harlow, PI 2011 – present, MaryFran Sowers, PI 1994-2011; Massachusetts General Hospital, Boston, MA – Sherri-Ann Burnett-Bowie, PI 2020 – Present; Joel Finkelstein, PI 1999 – 2020; Robert Neer, PI 1994 – 1999; Rush University, Rush University Medical Center, Chicago, IL – Imke Janssen, PI 2020 – Present; Howard Kravitz, PI 2009 – 2020; Lynda Powell, PI 1994 – 2009; University of California, Davis/Kaiser – Elaine Waetjen and Monique Hedderson, PIs 2020 – Present; Ellen Gold, PI 1994 - 2020; University of California, Los Angeles – Arun Karlamangla, PI 2020 – Present; Gail Greendale, PI 1994 - 2020; Albert Einstein College of Medicine, Bronx, NY – Carol Derby, PI 2011 – present, Rachel Wildman, PI 2010 – 2011; Nanette Santoro, PI 2004 – 2010; University of Medicine and Dentistry – New Jersey Medical School, Newark – Gerson Weiss, PI 1994 – 2004; and the University of Pittsburgh, Pittsburgh, PA – Rebecca Thurston, PI 2020 – Present; Karen Matthews, PI 1994 - 2020. NIH Program Office: National Institute on Aging, Bethesda, MD – Rosaly Correa-de-Araujo 2020 - present; Chhanda Dutta 2016- present; Winifred Rossi 2012–2016; Sherry Sherman 1994 – 2012; Marcia Ory 1994 – 2001; National Institute of Nursing Research, Bethesda, MD – Program Officers. Central Laboratory: University of Michigan, Ann Arbor – Daniel McConnell (Central Ligand Assay Satellite Services). Coordinating Center: University of Pittsburgh, Pittsburgh, PA – Maria Mori Brooks, PI 2012 - present; Kim Sutton-Tyrrell, PI 2001 – 2012; New England Research Institutes, Watertown, MA - Sonja McKinlay, PI 1995 – 2001. Steering Committee: Susan Johnson, Current Chair; Chris Gallagher, Former Chair. We thank the study staff at each site and all the women who participated in SWAN.

© 2022 Solomon DH et al. *JAMA Network Open.*
